# Is efficacy of miniscrew-assisted rapid palatal expansion in mid to late adolescents and young adults related to craniofacial sutures opening? A systematic review and meta-analysis

**DOI:** 10.4317/medoral.26935

**Published:** 2025-01-26

**Authors:** Aida Lázaro-Abdulkarim, Federico Hernández-Alfaro, Andreu Puigdollers-Pérez, Maria Giralt-Hernando, Basel Elnayef, Adaia Valls-Ontañón

**Affiliations:** 1 Department of Oral and Maxillofacial Surgery, School of Dentistry, Universitat Internacional de Catalunya, Barcelona, Spain; 2 Institute of Maxillofacial Surgery, Teknon Medical Center, Barcelona, Spain; 3 Department of Orthodontics and Dentofacial Orthopaedics, School of Dentistry, Universitat Internacional de Catalunya, Barcelona, Spain

## Abstract

**Background:**

Transverse maxillary deficiency is a relatively common type of malocclusion, that if left untreated will probably affect the permanent dentition. Recent investigations have proposed the use of bone-supported miniscrews around the midpalatal suture to expand the palate in late adolescents. The aim of this systematic review was to assess the efficacy of the Miniscrew Assisted Rapid Palatal Expansion (MARPE) technique in young adult patients, by quantifying skeletal expansion in relation to the age of the patient, as well as the impact upon other craniofacial sutures, and to describe the possible dental side effects.

**Material and Methods:**

An electronic and manual search was conducted, in which 17 were included in the study.

**Results:**

The estimated mean palatal opening width and nasal cavity width was 2.99 ± 0.33 mm and 2.24 ± 0.17 mm, respectively. A significant association was observed between midpalatal and pterygoid suture opening (*p*=0.010). No association was found between age and the MARPE technique (*p*=0.701).

**Conclusions:**

The results of this study show that the MARPE technique produces significant opening width in young adults at both at midpalatal suture and nasal cavity level, and apparently only significantly widens the pterygoid suture. Greater dental side effects are directly associated to a reduced midpalatal suture opening width.

** Key words:**Maxillary bone, cranial sutures, palatal expansion technique, bone screw, young adult.

## Introduction

Transverse maxillary deficiency is a relatively common type of malocclusion. If left untreated during the primary dentition, it leads to a narrow maxilla, deep palatal vault and posterior crossbite (1,2). Rapid palatal expansion (RPE) has been widely used for increasing the transverse dimensions of the maxilla in growing patients (1-5).

However, separation of the midpalatal suture becomes gradually more difficult with age (6), as ossification of the suture is a limiting factor for RPE (7-10). The process starts in the juvenile period and is fully completed around the third decade of life (7-9). Suture width in turn appears to decrease progressively throughout life (11). Therefore, in adult patients where calcification and interdigitation of the craniofacial sutures have already occurred, RPE becomes useless and undesired dental effects such as buccal tipping of the posterior teeth, decreased buccal bone thickness and buccal root resorption may result when tooth-borne devices are used (3,4,6-8,12). In order to avoid these adverse effects, surgically assisted rapid palatal expansion (SARPE) is the recommended procedure for increasing the transverse dimension of the maxilla in the adult patient (2). Several modifications have been proposed since Brown first described the technique in 1938, attending to where the osteotomies should be placed (13,14), and nowadays it is usually carried out under sedation, on an outpatient basis, and adopting a minimally invasive approach that implies low morbidity (15).

On the other hand, the Miniscrew Assisted Rapid Palatal Expansion (MARPE) expander is a modification of the conventional tooth-borne expander consisting of the incorporation of miniscrews into the palatal jackscrew (16-18). A pure skeletal expander has been designed to facilitate bicortical anchorage of the miniscrews within the cortical bone of the palate and the nasal floor (19). Several authors have demonstrated that this technique expands maxillary bone and increases nasal cavity width in mid to late adolescents and young adults without the necessity of osteotomies (2,20,21). Two different MARPE appliance designs have been described in the literature: bone-borne expanders, which comprise a palatal expander with four miniscrews; and hybrid expanders, which are a combination of tooth- and bone-borne devices (4).

However, there is controversy in the literature regarding the indicated patient age, since from a certain age the sutures of both maxillary buttresses are already ossified, and MARPE could imply a risk of breakage of other facial, skull base or cranial sutures (6). Moreover, while bone-borne expanders do not show dental movements, since no teeth are involved in the procedure (12,20,22), hybrid expanders have been related to buccal tipping of the anchored teeth and thinning of the buccal alveolar bone (3,23).

The aim of the study was to assess the efficacy of the MARPE technique in mid to late adolescents and young adults by quantifying palatal expansion at midpalatal suture level and nasal cavity width in relation to the age of the patient, as well as its impact upon other craniofacial sutures, and to describe the possible dental side effects related to the expansion device employed.

## Material and Methods

A systematic review was performed following the Preferred Reporting for Systematic Reviews and Meta-Analyses (PRISMA) standards and, after confirming that there were no similar studies registered in the International Prospective Register of Systematic Reviews (PROSPERO), it was registered on July 2020 under trial registration number CRD42020180556. This review extracted records from two main databases as well as from other sources (manual search), corresponding to articles written in English from September 2014 up to September 2024.

- Search strategy

The search was conducted in two main databases. The following Medical Subject Headings (MeSH) entry terms were used in the Medline database: ((“Palate” OR “Palates” OR “Hard Palate" OR “Hard Palates" OR “Palates, Hard” OR “Palatine Bone" OR “Bone, Palatine” OR “Maxilla" OR “Maxillas" OR "Maxillary Bone” OR “Bone, Maxillary" OR “Bones, Maxillary" OR “Maxillary Bones" OR “Maxillae” OR “Cranial Suture" OR "Suture, Cranial" OR "Sutures, Cranial” OR "Cavities, Nasal” OR “Cavity, Nasal" OR "Nasal Cavities”) AND (“Expansion Technique, Palatal” OR “Expansion Techniques, Palatal” OR “Palatal Expansion Techniques” OR “Technique, Palatal Expansion” OR “Palatal Expansion Technic” OR “Expansion Technic, Palatal” OR “Expansion Technics, Palatal” OR “Palatal Expansion Technics” OR “Technic, Palatal Expansion” OR “Maxillary Expansion” OR “Expansion, Maxillary” OR “Anchorage Procedure, Orthodontic” OR “Anchorage Procedures, Orthodontic” OR “Orthodontic Anchorage Procedure” OR “Procedure, Orthodontic Anchorage" OR “Procedures, Orthodontic Anchorage" OR “Orthodontic Anchorage Techniques" OR “Anchorage Technique, Orthodontic” OR “Anchorage Techniques, Orthodontic" OR “Orthodontic Anchorage Technique” OR “Technique, Orthodontic Anchorage” OR “Techniques, Orthodontic Anchorage” OR "Bone Screw" OR "Screw, Bone” OR "Screws, Bone” OR "Appliance Design, Orthodontic" OR "Appliance Designs, Orthodontic" OR "Design, Orthodontic Appliance" OR "Designs, Orthodontic Appliance" OR “Orthodontic Appliance Designs”) AND (“Adult, Young” OR "Adults, Young” OR “Young Adults” OR “Adult” OR “Adolescents” OR “Adolescence” OR “Teens” OR “Teen” OR “Teenagers” OR “Teenager” OR “Youth” OR “Youths” OR “Adolescents, Female” OR “Adolescent, Female” OR “Female Adolescent” OR “Female Adolescents” OR “Adolescents, Male” OR “Adolescent, Male” OR “Male Adolescent” OR “Male Adolescents”)). The following keywords were used in the Cochrane Library database: ((“Palate” OR “Maxilla”) AND (“Palatal Expansion Technique” OR “Bone Screw” OR “Dental Implants”) AND (“Adult” OR “Adolescent”)). It was based on the following PICO question: How does MARPE affect midpalatal and craniofacial sutures in young adults with transverse maxillary deficiency? Boolean operators (“OR” and “AND”) were combined with the search terms in order to identify any articles relating to palatal expansion techniques using miniscrews in young adults. Articles focused only in midpalatal suture opening width were not included (Table 1).

The records identified from each database were retrieved, and a manual search was made for additional publications.

- Records identification, screening and study eligibility

Two reviewers (A.L.-A. and A.V-O.) performed the electronic and manual searches. The screening process consisted of an initial assessment of all articles by title and abstract against the study eligibility criteria, followed by full-text evaluation. Any articles not fulfilling the inclusion criteria were excluded. Any disagreement between the two reviewers was resolved by consulting a third reviewer (F.H-A.). The kappa statistic (k) was used to evaluate the level of agreement between authors.

- Data extraction

Data was extracted and recorded independently by the two reviewers (A.L.-A. and A.V-O.) in a standardised Table. The following data was extracted: general information (authors, year of publication, study design), participants (groups, age and sex of patients, number of patients), expander device (design, activation protocol), radiographic evaluation of midpalatal suture opening width (and its shape, which depends on the level of opening), nasal cavity opening width, opening of other sutures, and dental/alveolar effects. Data were compared between authors, and any discrepancies were resolved by reviewing the study. If any data were missing in the study, the author of the article was contacted for further information.

- Assessment of heterogeneity and risk of bias

Heterogeneity was assessed by calculating the I2 statistical index (percentage of variability of the estimated effect that can be attributed to heterogeneity of the real effects) and evaluating the corresponding statistical test of nullity. Galbraith plots showed the degree of heterogeneity. In order to assess the risk of bias, funnel plots and the Egger test were used.

The quality of the papers was assessed using the adaptation of the bias analysis used by Haas *et al*. (24). The criteria based on sample selection, blinding of the authors, comparison between treatments, statistical analysis and outcome validation measured the degree of bias, definition of inclusion and exclusion criteria, and postoperative follow-up. The articles were categorised as presenting low risk if all the criteria were met; uncertain risk when only one criterion was missing; and high risk if two or more criteria were missing according to the analysis of Haas *et al*. (24).

- Statistical analysis

The meta-analysis was performed using R 3.5.1 (R Core Team 2013) (R core team: A language and environment for statistical computing. The R Foundation for Statistical Computing). The primary outcomes were midpalatal suture opening width, nasal opening width, opening of other craniofacial sutures, and dental/alveolar effects. In addition, an evaluation of potential confounding variables was also conducted, including the age of the subjects, the magnitude of expander activation and the type of expander device used. Meta-analysis consisted of an estimation of the proportion of midpalatal suture and nasal cavity weighted mean opening width of the included studies through a random-effects model. Meta-regression analysis was also performed to evaluate the potential effects of confounding variables through a random-effects model. For analysis of opening width (continuous variable), the weighted mean difference was used as a measure of overall size effect and effect at premolar (PM) and molar (M) level. Graphical representation was made by means of forest plots in all cases, with 95% confidence intervals (95%CI). The level of significance used in the analysis was 5% (α=0.05).

## Results

- Study selection

**Figure 1:**
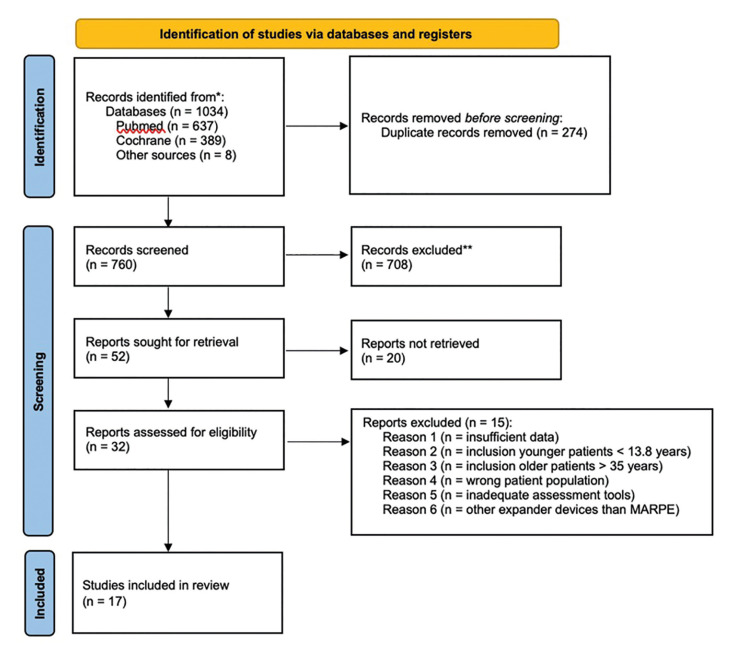
Flowchart of the systematic review.

The initial search yielded a total of 1034 articles. After title exclusion and abstract review, 32 articles were selected for the eligibility process and full-text reading. In addition, 8 articles were identified through manual searching. The level of inter-rater agreement was excellent (k = 0.883, 95% confidence interval = 0.831 - 0.920). Of these articles, 17 fulfilled the inclusion criteria and were included in the study (Fig. 1).

The inter-rater agreement coefficient was excellent (k = 0.913, 95% confidence interval = 0.847 - 0.951). As some of these studies had different subgroups depending on the used device (6,12,25-27), a total of 23 study samples resulted from the search and were therefore included in the qualitative analysis. Some studies moreover provided data segmented according to premolar (PM) or molar (M) position. Reasons for articles exclusion are summarized in Fig. 1.

**Figure 2:**
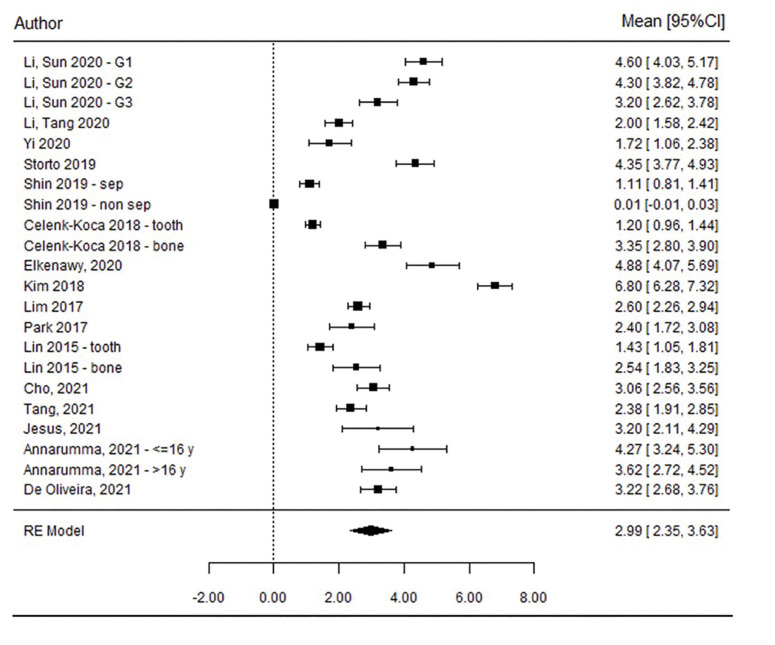
Forest plot of midpalatal suture opening width, TOTAL sample.

Twenty-two of the included studies provided midpalatal suture opening width data (2,3,6,12,21,23,25-34). The estimated mean opening width, in an overall sample size of 392 patients, was 2.99 ± 0.33 mm (95% CI 2.35 - 3.63, *p*<0.001) (Fig. 2). It therefore can be concluded that patients treated with MARPE obtained a statistically significant midpalatal suture opening width. Table 2 summarizes the characteristics of the included studies.

Since a very high heterogeneity between studies was detected (I2=98.7%, *p* <0.001), reporting results from 0 to 6 mm, the midpalatal suture opening width was evaluated at PM and M level separately. A mean opening width of 3.40 ± 0.39 mm (95% CI, 2.63 - 4.17) and 2.63 ± 0.34 mm (95% CI, 1.97 - 3.29) was obtained respectively, for the PM and M positions. Although greater opening width was obtained anteriorly than posteriorly, the heterogeneity remained high in both groups (PM: I2=94.7%, QH *p*<0.001; and M: I2=96.3%, QH *p*<0.001), suggesting heterogeneity was not induced by the level of measurement but was due to publication bias (*p*=0.005, Egger test).

**Figure 3:**
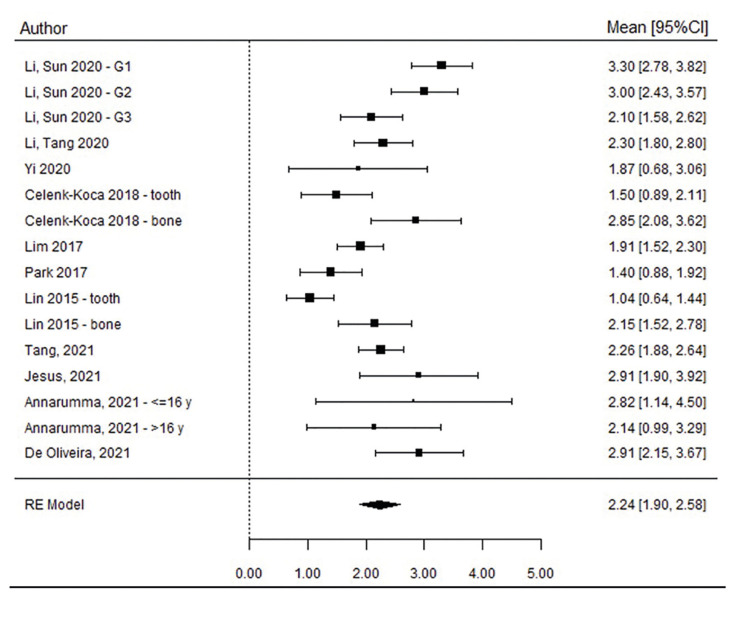
Forest plot of nasal cavity opening width, TOTAL sample.

When analysing the increased nasal cavity width from 16 studies (3,12,23,25-28,31-34) involving a total sample of 273 patients, the estimated opening width was 2.24 ± 0.17 mm (95% CI 1.90 - 2.58, *p*<0.001). Thus, it is estimated that MARPE produces a statistically significant nasal cavity opening width (Fig. 3).

The estimated mean opening width at PM and M level (3,12,25,28,32,33) was 2.14 ± 0.24 mm (95% CI, 1.67 - 2.62) and 1.86 ± 0.34 mm (95% CI, 1.19 - 2.53), respectively. Although important heterogeneity among studies was detected (I2=80.1%, *p* <0.001), the funnels plots revealed great symmetry, with no publication bias among the studies (*p*=0.289, Egger test).

The relationship between midpalatal suture and nasal cavity opening width was evaluated for the same 16 articles (3,12,23,25-28,31-34). A significant relationship was recorded (*p*<0.001), with a beta coefficient of 0.57, which means that for every 1 mm of midpalatal suture opening width, the nasal cavity width increased 0.57 mm. In turn, R2=82.3%, which means that most of the variability found in nasal opening could be directly explained by palatal opening. On evaluating this relationship separately at PM and M level, the association remained statistically significant (*p*<0.001), with a beta coefficient of 0.68 and 0.91, and R2= 100% and 62.9% at PM and M level, respectively.

Regarding the relationship between midpalatal suture opening and other craniofacial sutures, a significant association was observed between midpalatal and pterygoid suture opening (*p*=0.010) (3,26,28,30,31,34). There was clearly greater palatal suture opening width when there is a fracture of the pterygoid suture, with a beta coefficient of 1.46. In other words, for each mm of pterygoid suture separation, the midpalatal suture expanded 1.46 mm. When analysing the zygomatic and temporal sutures (3,26,28,30,31,34), no evidence of a significant association between the fracture of both sutures and midpalatal suture opening was observed (*p*=0.192 and *p*=0.287, respectively).

**Figure 4:**
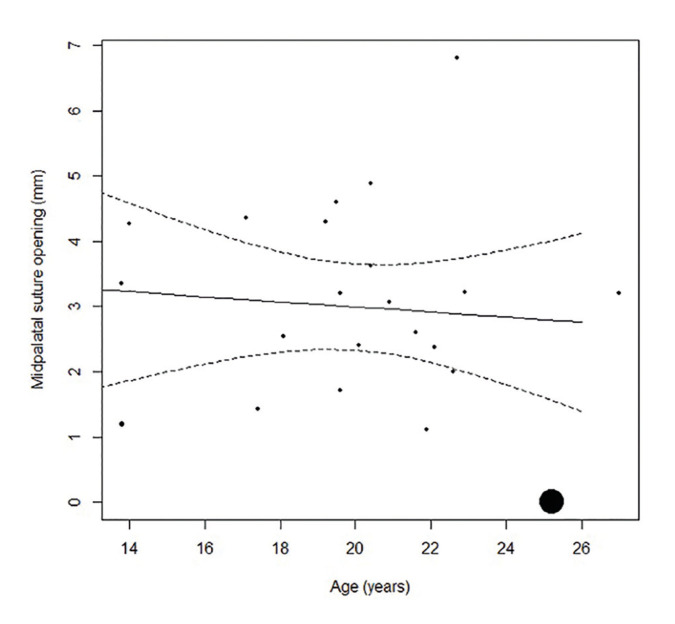
Relation between the midpalatal suture opening and age.

Meta-regression analysis contemplating age is of paramount importance, since age is considered a limiting factor for application of the MARPE technique, though the results showed no association between the two variables (*p*=0.701). Therefore, MARPE seems to be an effective technique in terms of midpalatal suture opening width in young adults (range from 13.9-29 years). In contrast, the subgroup of patients that achieved no expansion in the study published by Shin *et al*. (6) were a little older on average (25.17 ± 5.53, range: 18-36 years) (Fig. 4).

The analysis of the type of expander used showed tooth-bone-borne devices to be the most commonly used option (2,3,6,12,21,23,25-34), followed by three studies using bone-borne devices (12,25,28), and another two papers employing tooth-borne devices (12,25). The association between the type of expander and the amount of width gained is summarized in Table 3.

Overall, bone-borne and tooth-bone-borne devices produced significantly greater expansion than tooth-borne devices at both palatal and nasal level.

No relationship was found between the number of activations and the magnitude of palatine expansion achieved (*p*=0.892) (3,21,23,27,28,30,34).

Lastly, 15 of the included studies (> 50%) evaluated potential dental side effects. Although their relationship with the type of expander could not be assessed due to the lack of consistent information in the studies, the results of the meta-regression analysis showed greater dental side effects to be directly associated to a reduced midpalatal suture opening (*p*=0.021). On specifically evaluating dental buccal tipping (2,3,12,23,25-28,32,33), a tendency was observed towards reduced midpalatal suture opening (*p*=0.068). However, the magnitude of palatal expansion was not associated to the extent of buccal bone resorption (*p*=0.128). Meta-regression analysis between midpalatal suture opening and the thickness of the buccal and palatal plates, and the intercuspid distance could not be made, due to the lack of sufficient studies reporting this specific information.

Data reporting the risk of bias are shown in Table 4. The risk of bias of the papers included in this systematic review was classified as high in sixteen studies (2,3,6,14,21,23,25-34) and as medium in one study (12). The quality criteria related to these studies were related to the non-randomisation of the groups and the majority of treatments resolved in less than 12 months. Also, none of the studies reported blind assessment.

## Discussion

Our results based on 23 studies revealed that the MARPE technique produces significant opening width in young adults at both at midpalatal suture (2.99 ± 0.33 mm, *p*<0.001) and nasal cavity level (2.24 ± 0.17 mm, *p*<0.001), and apparently only significantly widening the pterygoid suture of all the studied craniofacial sutures.

However, as reflected in the literature (20,35-39), there is no consensus regarding the maximum patient age at which it can remain effective while not risking rupture of other distant craniofacial sutures (20,38). The results of our meta-regression analysis showed no significant association between age and expansion (*p*=0.701). So, as demonstrated by Grünheid (40), and Silva-Montero (10), the midpalatal suture density ratio should be considered as clinical predictor of skeletal response to MARPE instead of chronological age. On the other hand, although a limiting age where MARPE could work could not be established from this meta-analysis, the study of Shin (6) included a subsample aged 25.17 ± 5.53 years in which no expansion was obtained, and this could be taken as a reference.

On the other hand, the literature reports a wide range of opening magnitude of the maxillary suture after a MARPE procedure (from 0.9 to 6.8 mm) (3,6,14,21,23,28,29,31-33), which is probably due to the different maturation stages of the maxillary suture involved, but also of all other craniofacial sutures (32). There is strong evidence supporting the assumption that the MARPE technique secondarily produces the opening of other craniofacial sutures to some extent (23,30,31). Our results confirmed the correlation between midpalatal suture opening width and pterygopalatine suture breakage (*p*<0.05), though a quantitative correlation could not be established, since the studies only reported the presence or absence of suture opening, not its magnitude. In accordance with this, several clinical studies agreed that pterygopalatine disarticulation leads to greater midpalatal suture expansion (30,31,38). The included studies did not report secondary opening of the zygomatic and temporal sutures because they comprised patients under 30 years old, but previous studies have consistently recorded lateral displacement of these structures after MARPE in patients with mature midpalatal suture (3,26,29,31,34,39). However, it is important to note that fractures arising at the craniofacial sutures when using the MARPE technique exhibit an unpredicTable and uncontrolled pattern. Thus, although most publications do not report the complications derived from this technique, clinicians should be concerned about them, since some vascular and neural structures contained in the base of the skull could be damaged (38). Further research is therefore required to evaluate anatomical and biological features with a view to designing patient-tailored expansion protocols (20,38).

On the other hand, the reported results suggest that the expansion produced after MARPE is characterized by a V-shaped opening pattern, being wider anterior than posterior at the transverse plane (3.40 ± 0.39 mm versus 2.63 ± 0.34 mm, respectively) and greater palatine than nasal in the coronal plane (2.99 ± 0.33 mm at the palate versus 2.24 ± 0.17 mm in the nasal cavity). Our results also showed tooth-borne devices to produce significantly less nasal expansion (*p*=0.006). Regarding the transverse plane, the anterior palate has greater bone heights, allowing more sTable anchorage of the miniscrews, so the expander device is usually positioned more anteriorly (35). Cantarella *et al*. demonstrated that when the bone expander is placed in the posterior part of the palate, a more parallel split is facilitated (20). Moreover, the pterygopalatine suture limits the magnitude of expansion, especially in the posterior sector, which in the end dictates its opening pattern. Whilst several authors obtain a pyramidal opening (2,12,14,26,35), others describe a more parallel split (20,28,29), mostly related to the use of bicortical anchorage of the miniscrews (26,30,36,38). Similarly, greater expansion of the nasal cavity is obtained with 4-bicortical penetration expanders, than using monocortical miniscrew anchorage or hybrid devices (26,32,34). Regarding the V-shaped pattern in the coronal plane, it is probably due to two main reasons: firstly, since the naso-maxillary and zygomatic-maxillary buttresses are not cut as in SARPE procedures, they produce apical resistance; and secondly, with the exception of bone-borne expanders, the devices are mostly anchored to the palatal dental layer, resulting in greater expansion at this level.

However, regardless the magnitude and pattern of maxillary transverse augmentation obtained after the MARPE technique, it is not exempt from relapse. Tang *et al*., in 2021, in a study based on 31 patients between 18-33 years of age, recorded a suture width increase from 0.12 to 2.50 mm, though this was followed by a decreased to 0.75 mm after one year of retention - corresponding to 70% of the total expansion achieved. The authors speculated that this relapse could have been secondary to bone remodeling (31).

To some extent, the MARPE technique is associated to dental side effects. The most commonly reported effects are dental buccal tipping of the anchored teeth, decreased buccal bone thickness, and vertical bone loss (3,12,23,26). This study showed that greater dental side effects occurred more frequently when a reduced midpalatal suture opening width was obtained (*p*<0.05). In other words, when maxillary suture could not be opened, the anchored teeth were overload and, consequently, dental side effects appeared. Due to the limited articles and the heterogeneity of the measurements, tooth inclination could not be quantified in this study, though there was a tendency towards increased buccal dental tipping of the anchor teeth associated to reduced midpalatal suture opening (*p*=0.068).

With regard to buccal bone resorption, and unlike what would be expected, this study failed to demonstrate any association between the extent of palatal expansion or type of expander used and buccal bone loss of the anchor teeth (*p*=0.128). In contrast, several studies recorded a significant decrease in buccal bone thickness (3,23), especially at the level of the first molars. In order to avoid the abovementioned dental side effects, bone-borne devices are preferable.

The present meta-analysis has several limitations, such as the important clinical heterogeneity among the studies, which jeopardized the statistical analysis, and the low quality of evidence of the included studies, due to their retrospective design, methodological issues, and different follow-up periods. Furthermore, most publications did not include data on patients with MARPE failure, i.e., without midpalatal suture opening, excluding them from the study (37). This can be associated to some risk of bias when analysing the success of the MARPE technique. It is important to note that the majority of publications regarding this topic are retrospective studies based on convenience populations, with a small sample size, and a lack of correlation between chronological age and midpalatal suture density ratio. Thus, further investigations in the form of prospective randomised studies with larger sample sizes are required in order to draw firm conclusions.

Our results show that MARPE technique produces a significant opening width in young adults at both the midpalatal suture and nasal cavity levels, and apparently only significantly widening the pterygoid suture of all studied craniofacial sutures. Greater dental side effects are directly associated to reduced midpalatal suture opening width, which in turn is linked to tooth-borne devices. The results of the present study should be interpreted with caution, and further research is recommended, preferably in the form of randomised controlled clinical trials involving powerful samples and long-term follow-up times.

## Figures and Tables

**Table 1:** Eligibility criteria.

Category	Inclusion Criteria	Exclusion Criteria
Study design	Interventional studies	Case report Review of the literature Ex-vivo studies
Population (P)	Young adult patients (13.8 - 30 years) Transverse maxillary deficiency Minimum sample size (n = 10)	Young patients (< 13.8 years) Adult patients (>30 years) Craniofacial disorder
Intervention (I)	MARPE	Other expansion treatments
Control (C)	No treatment	-
Outcome (O)	Effects on midpalatal and craniofacial sutures Dental and alveolar effects Cone Beam Computed Tomography evaluation	-
Others	Articles published in the last 10 years	Other languages than English or Spanish No full text available

**Table 2:** Characteristics of the included studies.

Author, year	Type study	Groups	Nº patients	Mean age patients (interval)	Expander design	Midpalatal suture opening width			Shape widening
						Total (mm)	Premolar	Molar	
Lin, 2015(25)	R	Tooth-borne	28	17.4 ± 3.4	TB	NR	1.71 ± 0.92	1.14 ± 0.47	Pyramidal
		Bone-borne		18.1 ± 4.4	BB	NR	3.08 ± 1.63	1.99 ± 1.18	
Choi, 2016 (14)	R	NA	20	20.9 ± 2.9 (18 - 28)	TBB	2.24	NR		Triangular
Park, 2017 (3)	R	NA	14	20.1 ± 2.4 (16 - 26)	TBB	2.4 ± 1.3	NR		Parallel, pyramidal
Lim, 2017 (23)	R	NA	24	21.6 ± 3.1 (18.25 - 26.75)	TBB	2.60 ± 0.85	NR		Parallel, pyramidal
Kim, 2018 (21)	R	NA	14	22.7 ± 3.3 (18.3 - 26.5)	TBB	6.8 (4.8 - 8.8)	NR		NR
Celenk-Koca, 2018 (12)	P	Tooth-borne	20	13.84 ± 1.36	TB	NR	1.3 ± 0.7	1.1 ± 0.4	Triangular
		Bone-borne	20	13.81 ± 1.23	BB		3.6 ± 1.2	3.1 ± 1.3	
Shin, 2019 (6)	R	Separation	25	21.88 ± 4.91	TBB	0.90 ± 0.81	1.11 ± 0.76		NR
		Non-separation	6	25.17 ± 5.53			0.01 ± 0.02		
Storto, 2019 (2)	NR	NA	20	17.1	TBB	NR	4.7 ± 1.49	4.0 ± 1.17	Triangular
Li, 2020 (26)	R	4-all-bicortical	17	19.5 ± 3.1 (15.1-24.5)	TBB	4.6 ± 1.2	NR	NR	Pyramidal
		2-rear-bicortical	17	19.2 ± 3.5 (15.5-25.6)		4.3 ± 1.0	NR	NR	
		Non-4-bicortcal	14	19.6 ± 3.5 (15.7-24.8)		3.2 ± 1.1	NR	NR	
Li, 2020 (34)	R	NA	22	22.6 ± 4.5	TBB	2.0 ± 1.0	NR	NR	NR
Elkenawy, 2020 (29)	R	NA	31	20.4 ± 3.2 (17 - 27)	TBB	NR	4.98 ± 1.94	4.77 ± 2.65	Parallel
Yi, 2020 (28)	R	NA	13	19.61 ± 5.25 (15 - 29)	BB	NR	2.19 ± 1.72	1.25 ± 0.69	Triangular
Cho, 2022 (30)	R	NA	23	20.9 ± 3.65 (16 - 27)	TBB	3.06 ± 1.23	3.04 ± 1.13	2.52 ± 1.33	Triangular
Tang, 2021 (31)	R	NA	31	22.14 ± 4.76 (18 - 33)	TBB	2.38 ± 1.33	NR	NR	Pyramidal
Jesus, 2021 (32)	R	NA	12	15-39	TBB	3.20 ± 1.92	NR	3.20 ± 1.92	Parallel
Annarumma, 2021 (27)	R	< 16 years	11	13.96 ± 1.82	TBB	NR	5.00 ± 1.84	3.54 ± 1.65	Triangular
		> 16 years	13	20.43 ± 3.81			4.40 ± 1.51	2.84 ± 1.80	Triangular
de Oliveira, 2021 (34)	R	NA	17	22.9 (15 - 37)	TBB	NR	3.69 ± 1.42	2.75 ± 0.85	Parallel

NA: Not applicable; NR: Not reported; R: retrospective; P: prospective; TB: tooth borne, BB: bone borne, TBB: tooth-bone borne.

**Table 3:** Results of the meta-regression of the suture opening width according to the type of expander.

Suture	Midpalatal suture opening width					Nasal cavity opening width					
	Beta	SE	IC 95%	Z (*p-value*)	R^2^	Beta	SE	IC 95%		Z (*p-value*)	R^2^
Tooth-bone (ref.)	-	-	-	0.392	-	-	-	-	-	0.085	-
Tooth-borne	-1.81	1.32	-4.40 0.78	0.171	0.0%	-1.07	0.50	-2.06	-0.09	0.032*	29.0%
Bone-borne	-0.37	1.14	-2.60 1.86	0.746		0.01	0.48	-0.93	0.94	0.989	

*p<0.05; **p<0.01; ***p<0.001.

**Table 4:** Quality assessment of the included studies.

Study	Randomi-zation	Comparison between treatments	Blind assessment	Validation of measurements	Statistical analysis	Defined inclusion/ exclusion criteria	Report of follow-up (at least 12 months)	Risk of bias
Lin (25)	No	Yes	No	Yes	Yes	Yes	No	High
Choi (14)	No	No	No	No	Yes	Yes	No	High
Park (3)	No	No	No	Yes	Yes	Yes	No	High
Lim (23)	No	No	No	Yes	Yes	Yes	Yes	High
Kim (21)	No	No	No	Yes	Yes	Yes	Yes	High
Celenk-Koca (12)	Yes	Yes	No	Yes	Yes	Yes	No	Medium
Shin (6)	No	No	No	Yes	Yes	Yes	No	High
Storto (2)	No	No	No	No	Yes	Yes	No	High
Li, Sun (26)	No	Yes	No	Yes	Yes	Yes	No	High
Li, Tang (34)	No	No	No	Yes	Yes	Yes	No	High
Elkenawy (29)	No	No	No	Yes	Yes	Yes	No	High
Yi (28)	No	No	No	Yes	Yes	Yes	No	High
Cho (30)	No	No	No	Yes	Yes	Yes	No	High
Tang (31)	No	No	No	Yes	Yes	Yes	No	High
Jesus (32)	No	Yes	No	Yes	Yes	Yes	No	High
Annaruma (27)	No	Yes	No	No	Yes	Yes	No	High
de Oliveira (33)	No	Yes	No	Yes	Yes	Yes	No	High

Risk of bias assessment: High: 0 to 4 Yes - Medium: 5 or 6 Yes - Low: 7 Yes.
